# A Qualitative Analysis of Disclosure Patterns among Women with Sexual Violence-Related Pregnancies in Eastern Democratic Republic of Congo

**DOI:** 10.1371/journal.pone.0164631

**Published:** 2016-10-14

**Authors:** Monica Adhiambo Onyango, Gillian Burkhardt, Jennifer Scott, Shada Rouhani, Sadia Haider, Ashley Greiner, Katherine Albutt, Colleen Mullen, Michael VanRooyen, Susan Bartels

**Affiliations:** 1 Boston University School of Public Health, Department of Global Health, Boston, Massachusetts, United States of America; 2 Harvard Humanitarian Initiative, Cambridge, Massachusetts, United States of America; 3 Boston University School of Medicine, Department of Obstetrics and Gynecology, Boston, Massachusetts, United States of America; 4 Beth Israel Deaconess Medical Center, Department of Obstetrics and Gynecology, Boston, Massachusetts, United States of America; 5 Brigham and Women’s Hospital, Division of Women’s Health, Boston, Massachusetts, United States of America; 6 Harvard Medical School, Boston, Massachusetts, United States of America; 7 Brigham and Women’s Hospital, Department of Emergency Medicine, Boston, Massachusetts, United States of America; 8 Department of Obstetrics and Gynecology, University of Illinois at Chicago, Chicago, Illinois, United States of America; 9 Beth Israel Deaconess Medical Center, Department of Emergency Medicine, Boston, Massachusetts, United States of America; 10 Massachusetts General Hospital, Department of Surgery, Boston, Massachusetts, United States of America; 11 Boston Medical Center, Department of Psychiatry, Boston, Massachusetts, United States of America; 12 Harvard School of Public Health, Boston, Massachusetts, United States of America; 13 Queen’s University, Department of Emergency Medicine, Kingston, Canada; Örebro University, SWEDEN

## Abstract

The Democratic Republic of the Congo (DRC) has experienced nearly two decades of civil conflict in the Eastern regions of North and South Kivu. This conflict has been notorious for the use of sexual violence as a weapon of war, leading in many cases to pregnancy after rape. The objectives of this analysis were: 1) to describe patterns of sexual violence-related pregnancy (SVRP) disclosure; 2) to consider why survivors chose to disclose to particular individuals; and 3) to examine the dialogue around SVRPs between women with SVRPs and their confidants. In South Kivu Province, Democratic Republic of Congo, two sub-groups of sexual violence survivors completed qualitative interviews, those currently raising a child from an SVRP (parenting group, N = 38) and those who had terminated an SVRP (termination group, N = 17). The findings show that a majority of SVRPs were conceived when participants were held in sexual captivity for prolonged periods of time. The SVRPs were disclosed to friends, family members, other sexual violence survivors, community members, spouses, health care providers, or perpetrators. The confidants were most often chosen because they were perceived by the participants as being discreet, trusted, and supportive. The confidants often provided advice about continuing or terminating the SVRP. Trust and discretion are the most important factors determining to whom women with SVRPs disclose their pregnancies. The vital role of confidants in giving support after disclosure cannot be overlooked. Providing opportunities for survivors to safely disclose their SVRPs, including to health care providers, is a necessary first step in allowing them to access safe and comprehensive post-assault care and services.

## Introduction

Sexual violence has been prevalent during two decades of armed conflict in eastern Democratic Republic of Congo (DRC). [1] Sexual violence in eastern DRC is frequently characterized by multiple perpetrators and repeated assaults on victims while being held in captivity.[2,3] The nature of these sexual assaults increases the risk of unintended pregnancies and up to 17% of sexual violence survivors in eastern DRC are estimated to have a resultant sexual violence-related pregnancy (SVRP).[4] With limited access to comprehensive reproductive health services in settings such as DRC, unintended pregnancies are thought to be associated with increased risks to women and poor pregnancy outcomes.[5–9]

Previous studies in eastern DRC suggest that women with sexual violence-related pregnancies (SVRPs) also face a high burden of psychosocial consequences such as spousal abandonment [10,11], social rejection[12], negative socioeconomic impact [13] and psychological symptoms.[11,13] Stigma following sexual violence has previously been documented in eastern DRC,[14–15] and may impact disclosure of a sexual violence-related pregnancy (SVRP) in several important ways. Stigma may prevent survivors from disclosing that the pregnancy was conceived from sexual violence and influence who is informed of the pregnancy, resulting in delayed disclosure and/or failure to seek pregnancy-related care, and may influence women’s decisions to continue or to terminate the pregnancy [16]. Termination of pregnancy in DRC is highly restricted, permissible only to save the life of a woman,[17,18] and access to skilled providers for termination services is limited [19], which may further impact disclosure of SVRPs, especially among survivors considering pregnancy termination.

Much of the related literature focuses on disclosure of sexual assaults (as opposed to SVRPs) and comes from Western populations. For instance, research has shown that sexual violence survivors are more likely to disclose to formal entities including law enforcement and health care providers, when the assault is perpetrated by a stranger or use of a weapon. [20] College women in the United States overwhelmingly (86%) tend to disclose to a female peer. [21] It has also been documented that reactions perceived as being negative by the survivor upon disclosing a sexual assault can be detrimental to women’s adjustment following trauma [22, 23] and that non-disclosure is associated with higher symptoms of depression and post-traumatic stress disorder. [23] Less is known about disclosures of SVRPs although some U.S. based studies have found that approximately one half of obstetrical practices screen for pregnancy resulting from sexual violence. [24] Although less than one third of SVRPs were disclosed as a result of screening, [25] some authors have advocated for more widespread screening for SVRPs and for creating an environment more conducive to disclosure as a way to alleviate some of the survivors’ stress. [26]

Even less is known about disclosure of SVRPs in conflict or post-conflict settings where access to health care is often restricted, resources for post-sexual assault care are limited and stigma surrounding sexual violence is high.[14–15, 27, 28] Evidence on disclosure patterns in this context is important to informing our understanding of access to care, decision-making around keeping versus terminating SVRPs, mental health outcomes and experiences of stigma and rejection. An improved understanding of these disclosure-related dimensions could foster programmatic and policy changes that facilitate disclosure in safer environments and advocate for the unique needs of sexual violence survivors with SVRPs in DRC and similar post-conflict settings. To help address the existing knowledge gap, this paper presents qualitative data from a larger mixed methods study that examined outcomes of SVRPs in South Kivu Province, DRC. [26, 29, 30] The objectives of this analysis were: 1) to describe patterns of SVRP disclosure; 2) to consider why survivors chose to disclose to particular individuals; and 3) to examine the dialogue around SVRPs between women with SVRPs and their confidants.

## Materials and Methods

### Participant Recruitment

The data presented here is derived from a mixed methods study conducted in Bukavu, South Kivu Province from October to November, 2012. The study was designed to assess women reporting SVRPs who were currently raising a child from an SVRP (parenting group) and women who had terminated an SVRP (termination group). Respondent-driven sampling (RDS), a peer-recruitment method designed to sample hard-to-reach populations, was used to recruit a total of 852 participants to complete a quantitative questionnaire. Every twentieth participant in the parenting group and every fifth participant in the termination group also completed a qualitative interview. Although respondent-driven sampling (RDS) was used to recruit participants, the qualitative data represent a convenience sample due to smaller sample sizes. Quantitative data [26, 30, 16] and complete study methods, including respondent-driven sampling (RDS) methodology [30], have been published previously. Qualitative data on disclosure of SVRPs among both the parenting and termination groups are presented here.

#### Inclusion and Exclusion Criteria

Women who self-identified as survivors of sexual violence since the start of the war (~1996), became pregnant as a result of sexual violence, and were aged 18 years or older were identified by local partner organizations for inclusion in this study. The parenting group included women who had delivered a live born infant as a result of an SVRP and were living with and raising the child at the time of the study. Women were excluded from this group if they reported a stillbirth, if the child had since died, or if the child was not living with or in the care of the mother at the time of the study. Participants who self-reported termination of an SVRP were included in the termination group. Women with SVRPs that reported spontaneous abortions were excluded. Women with a history of more than one SVRP were eligible for both the parenting and termination groups.

### Qualitative Methods

Qualitative questionnaires designed for each study group were comprised of semi-structured questions on pregnancy history, sexual violence and experiences related to the SVRP, disclosure of the SVRP, and decision-making process regarding continuing or terminating the SVRP [S1–S4 Files.] The guiding questions included examples of probes to be used by interviewers as follow up to specific questions. The questionnaires were written in English, translated into Kiswahili by a local translator, and then back translated by a different translator. A third translator and a local panel of research collaborators resolved any differences in translation of the study questionnaires to ensure fidelity to the meaning of the questions. Local interviewers were selected based on their expertise and sensitivities in the subject matter and trained in qualitative research methods. Individual in-depth interviews were conducted in Kiswahili in a private setting and responses were hand recorded in Kiswahili by the interviewer. On average the qualitative interviews lasted approximately 30 minutes.

Transcripts of the interviews were translated into English by a local translator and electronic files were created and subsequently uploaded to the qualitative data analysis software Dedoose [31] (Version 5.0.11, Los Angeles, CA). Transcripts were reviewed line by line to identify thought patterns, feelings and actions captured in the study interviews and to determine preliminary coding structures for thematic organization of the data. Two researchers then independently coded each transcript. The data were subsequently organized into key conceptual themes and grounded theory tools of constant comparison, theoretical sensitivity and triangulation of researchers were used as the analytic approach. [32, 33] Care was also taken in maintaining sensitivities to the existing literature. Coding inter-rater reliability, measured with a pooled Cohen’s kappa, was 0.92. [34]

### Ethical Considerations

The institutional review board at Harvard School of Public Health approved this study and a community advisory board provided study oversight in Bukavu. The Medical Inspector in South Kivu provided permission to conduct the study. No identifying information was collected from study participants and verbal informed consent was obtained prior to enrollment. If the participant agreed to take part in the study after having the informed consent explained and after having an opportunity to ask questions, consent was indicated by checking a box on the electronic quantitative survey. Participants were offered a headscarf ($1 USD) as compensation for their time and transportation reimbursement (up to $8 USD return) was provided directly to taxi drivers. Participants also received a referral card for medical care and/or mental health counseling and a trained psychosocial assistant was available during all interviews to provide assistance to any participants who were distressed or who requested counseling during or after the interview.

## Results

### Demographics of Participants

A total of 38 and 17 interviews were completed with participants in the parenting and termination groups, respectively. The mean age of all 55 respondents was 33.5 (18–60 years). The total number of pregnancies per woman ranged from 1–12 (mean 4.6) and the number of living children per woman at the time of the survey ranged from 1–9 (mean 3.9). Many women reported they were divorced or separated from their spouses as a result of sexual violence (16/55). Participants also identified as widowed (13/55), married (11/55), single or never married (11/55) or reported their husbands missing (4/55). Some women in the married group also reported their husbands were kidnapped and were unsure if their husbands were alive at the time of the study.

### Theme 1: Choice of confidants and reasons for disclosing to particular individuals

Participants were asked to whom they had first disclosed the SVRP and if they subsequently told anyone else about the SVRP. Women in both study groups reported disclosure to friends or community members, typically described as a *neighbor* or ‘*wise’ woman* in their villages or occasionally a *pastor* or a *local chief*. Women who disclosed to family members were primarily in the parenting group and the majority of this group chose to disclose to their *mothers*. Women also reported disclosure to other family members, including *mothers-in-law*, *aunts*, *uncles*, *grandmothers*, *daughters*, *cousins* and *fathers-in-law*. The majority who disclosed to their spouses terminated the pregnancies with only one woman from the parenting group reporting having disclosed to her spouse. Overall, participants in this study gave considerable thought to who they confided in about their SVRPs.

#### Trust in the confidant

There were several reasons given for disclosing the SVRP to specific individuals. Regardless of whether the confidant was a family member, neighbor, or spouse, women in both the parenting and termination groups chose their confidant because they believed this individual was discrete and could be trusted not to disclose to others.

“When I realized I was pregnant, the first person I informed was my close friend. I chose her because she was very discreet.”

20-year-old woman, not married and raising a child born from an SVRP

“I informed my friend because she was discreet. She didn’t disclose it.”

25-year-old woman, abandoned by her spouse who terminated an SVRP

''I let my spouse know… because he was the person I trusted. I believed we could decide together and sort the issue out. He asked me to find a solution to my problem.”

24-year-old woman, married and who terminated an SVRP

Participants also indicated that they chose to disclose to a particular individual because they were close to him / her and because they were confident that they would be comforted and supported by this person.

“I informed first my mother. I chose my mother because I believed she would understand me and provide some advice.”

36-year-old woman, not married and parenting a child born from an SVRP

“I informed my neighbor… I only chose to inform her because she was close to me.”

37-year-old women, abandoned by her spouse and raising a child born from an SVRP

Among the women who did not disclose to anyone, lack of trust was cited as a reason for not disclosing the SVRP. One woman who did not disclosure the pregnancy reported moving to another location where she believed she could more easily conceal that it was an SVRP.

“Since I didn’t trust anyone, I couldn’t disclose this information…. Nobody asked me a question [about the pregnancy] because no one was informed.”

36-year-old woman, widowed, who terminated an SVRP

“I didn’t inform anybody. So nobody knew I was coming from the bush where I had met the [armed group]. I took refuge in the territory…where I was not known.”

48-year-old woman whose spouse was kidnapped and was raising a child born from an SVRP

#### Survivor of sexual violence as confidant

Women who experienced sexual captivity by armed combatants often met other women while in captivity and participants reported that they chose to disclose the SVRP to another survivor of sexual violence because they trusted the woman or felt that the other survivors had had a similar experience and might be able to provide advice.

“*I informed one of the five women with whom we were taken to the bush…I informed my bush mate with whom we succeeded to escape from the bush*.*”*

33-year-old woman, separated from her spouse, raising a child from an SVRP

*“The first person I informed was a woman with whom we were taken to the bush*. *I chose her because I wanted to know what to do*.*”*

25-year-old woman separated from her spouse, raising a child form from an SVRP

In some cases, the woman disclosed to another survivor who had also become pregnant as a result of sexual violence.

“*I informed that friend because she was also pregnant from the same combatant*. *She asked me to think before deciding on termination or not*.*”*

52-year-old woman, married and who had terminated an SVRP

#### Perpetrators as confidants

Some women reported that they informed the sexual violence perpetrator about the pregnancy. A subset of these women reported they were ridiculed by the perpetrators while another participant reported that she was beaten upon disclosing the pregnancy. Other participants reported that the perpetrators simply did not care that they were pregnant.

“It was two months later when I noticed I didn’t have my period. When I realized the pregnancy was from sexual violence, I felt ill. I informed my partner in the bush, but he didn’t care.”

20-year-old woman, not married and raising a child born from an SVRP

“When I informed the [combatants] that I was pregnant they were happy and some of them were laughing at me. One of the [combatants] suggested raping me and then showing me the way to escape. I accepted.”

37-year-old woman, married and who terminated an SVRP

“When I informed the [combatants] I was feeling weak. I was beaten.”

26-year-old woman, not married and raising a child born from an SVRP

#### Health care providers and traditional healers as confidants

A few women had informed a health care professional about the SVRP or a traditional healer. In such cases, women often discovered that they were pregnant after seeking care at a local health facility or while they were in the hospital for other non-pregnancy related injuries.

“*I went to see a nurse and he told me I was pregnant*. *I underwent medical treatment until delivery*.*”*

48-year-old woman, separated from her spouse, raising a child from an SVRP

“*When I realized I was pregnant I told the woman who was looking after me at the hospital*. *I also informed the nurse who was treating me*.*”*

40-year-old woman, hospitalized for a leg injury from the [combatants] raising a child from an SVRP

Women in the termination group who sought out a medical provider, stated that they chose to do so in part because they felt the provider was discreet or they wanted to ensure they received good medical care again highlighting how themes of discretion and trust in the confidant may have influenced disclosure patterns.

“I went to see the nurse, I didn’t inform anybody because I have another child resulting from sexual violence, and I didn’t want anybody else to know apart from the nurse. I knew the nurse was discreet. When I told him that I didn’t like it and asked him to terminate the pregnancy he said nothing.”

21-year-old woman, not married, who terminated an SVRP

“I chose the nurse because he could provide good medical care.”

33-year-old woman, whose husband was killed by [combatants] and who terminated an SVRP

### Theme 2: Dialogue around SVRPs and advice of the confidant

In further probing why women chose to disclose to certain individuals, information about the content of discussions with confidants also emerged. When describing why a confidant was chosen, women in both groups often confided in individuals whom they thought would know what to do, in particular in relation to continuing or aborting the pregnancy, and sought and relied on the advice of their confidants.

“I chose the wise women because of her age and many women needed her assistance for such issues. She didn’t inform anybody else. I didn’t expect to get immediately medicine to terminate the pregnancy but I wanted first to get advice from her.”

30-year-old woman, whose husband was kidnapped by [combatants] and who terminated an SVRP

In many instances, women went on to explain how the advice from their confidants ultimately influenced their decision to either continue or terminate the pregnancy.

“She [friend] advised me to terminate it as she did…. I know what I did was illegal and I could be arrested if the information were disclosed.”

40-year-old woman, married and who had terminated an SVRP

“I informed my mother….She was so sad and started weeping. She advised me not to perform termination.”

32-year-old woman, separated and raising a child from an SVRP

While in other situations, the woman decided to go against the advice she received from her confidant.

“The first person I informed was my mother. She asked me to inform my mother-in-law as well. The mother-in-law asked me to perform a termination but I objected.”

25-year-old woman, married and raising a child born from an SVRP

Following disclosure of the SVRP, discussion between the woman and her confidant(s) often turned to religion and/or innocence of the unborn child.

“I leaned on the door when my neighbor asked me to keep the child. She said, ‘this child is innocent and he is like others. He is a child and shouldn’t suffer…she was concerned by my problem. She often takes me to hospital when it is necessary. She asked me to pray and keep the pregnancy and not to complain.”

40-year-old woman, widowed and raising a child born from an SVRP

“I wanted to terminate the pregnancy but my aunt and the elder sister of my late mother advised me not to terminate it. [They] told me the child born from sexual violence was also created by God as any child. Then I decided to carry it to full term.”

35-year-old woman, separated and raising a child born from an SVRP

Potential social consequences, such as spousal rejection, were common themes that emerged among women who disclosed to their spouses. Of the seven women who informed their spouses about the SVRP, six reported that they were asked or pressured to terminate the pregnancy and/or rejected by their spouse.

“When my spouse was informed he rejected me….my concern is that my life is very bad. I live without a spouse now. I live in the neighbor’s family. I make use of my sex to get money.”

37-year-old woman, abandoned by her spouse and raising a child born from an SVRP

“I informed my husband about the pregnancy from sexual violence. I informed my spouse because we were living together. He was so angry he asked me to learn from other women how to perform a termination.”

27-year old woman married who terminated an SVRP

One woman who disclosed the pregnancy to her spouse was later abandoned by him after deciding to continue the pregnancy to term.

“*I was so restless that I wanted to attempt suicide*. *I began seeking how to terminate that pregnancy but I failed…*. *Unfortunately*, *my spouse learned about the pregnancy and rejected me*.*”*

37-year-old woman, abandoned by her spouse raising a child born from an SVRP

## Discussion

These qualitative data highlight that sexual violence survivors with SVRPs may disclose the pregnancy to a variety of personal contacts, including friends, family members, neighbors, highly regarded community members, other survivors of sexual violence or the sexual violence perpetrators. The most important considerations influencing whom to inform were discretion and trust, feeling close to the individual, knowing that the individual would be supportive, and a perception that the confidant would know what to do in the situation. Some confidants advised the participant to carry the pregnancy to term while others advised the participant to terminate the pregnancy.

Confidentiality was a particular theme throughout and should be considered in the context of previous research on stigma among sexual violence survivors and women with SVRPs in eastern DRC. [14–16] Many women in this sample disclosed to a trusted female peer, either a family member, friend, or community member and this is in keeping with what has been described among U.S. college students. [21] Within this dataset there were few reports of participants experiencing stigma or rejection from female peers and few participants reported a negative response from their confidants. We hypothesize that this may have been because women in the current study were very selective in whom they disclosed to. Alternatively, it’s possible that we simply failed to capture experiences of negative responses from female peers with this survey. The adverse effects of a negative response after disclosing a sexual assault have been well documented in the North American context. [22, 35, 36] Further research is needed to better understand the impact of negative responses after disclosure in settings of conflict related sexual violence such as DRC.

In a report on children conceived from sexual violence in North Kivu Province, DRC Liebling described her observation that older women were less likely to disclose the pregnancy.[13] Although not elaborated on in the report, this may be because older women were more likely to be married and were able to conceal the pregnancy as arising from marital relations. In our data, of the three participants who reported not disclosing the SVRP to anyone, two were widowed at the time of the survey and one reported that her husband was missing. It is unclear whether there is a relationship between marital status and the decision to not disclose the pregnancy in these instances and this may warrant future research.

Women who disclosed to their spouses were either asked to terminate or were rejected by their spouses as a result of sexual violence and/or the SVRP. It should be noted however, that currently married women were under-sampled in the qualitative parenting survey in comparison to the quantitative parenting survey (5% versus 32%). It is unknown how this could affect the results as we may have failed to capture married women who had disclosed to their spouses and were still married while raising the child born from an SVRP.

A number of women who were in captivity reported disclosure of the SVRP to the perpetrator. These participants did not provide a specific reason for disclosing to the perpetrator although there are several hypotheses. First, women may have hoped the perpetrator would release them from captivity upon learning about the pregnancy. Second, women may have hoped that the perpetrator would provide some assistance with the pregnancy, such as access to medical care. Or finally, some women may have hoped that the perpetrator would provide support in raising the child.

In this sample, it was rare for women to disclose SVRPs to health care providers and although the context is very different, this finding is similar to that reported by North American researchers who have found that assault survivors were more likely to disclose to informal support providers such as friends and family than to health care providers. [21] However, there are other potential explanations specific to DRC, particularly for women considering terminating the pregnancy, including the complex Congolese legal statue for abortion services. The 1982 DRC Penal Code, stipulates that abortions are illegal and subject to five to fifteen years imprisonment except in cases of medical “necessity” to save the life of the woman.[6] It would be natural for women considering pregnancy termination to be concerned about facing legal consequences and for this to impact disclosure of the SVRP. However, only two women mentioned fear of facing legal consequences during their qualitative interviews, both of whom had terminated SVRPs making it unlikely that this was the major reason for lack of disclosure to health care providers. An alternate potential explanation specific to the DRC is that many women in this context have limited access to medical care, either because of financial constraints, insecurity, or lack of human resources within the health care system. Therefore, it is also possible that women in the current study failed to disclose to health care providers because they did not have access to a health care.

In general, early disclosure of sexual assaults to formal support providers is recommended as it allows for evidence collection and for prompt emergency medical care.[37] One of the most time sensitive medical interventions is emergency contraception (in addition to HIV prophylaxis). However, the women in this sample were already pregnant as a result of sexual violence and the study was intended to look at disclosure of SVRPs rather than sexual assaults. If there was perception that the health care system had little to offer women with SVRPs and if disclosure to a health care provider carried risk of a negative reaction, breach of confidentiality, stigma and rejection, it is understandable why women may have chosen not to disclose to health care personnel. Since most women who terminated SVRPs in this study were able to do so outside of the formal health care system by using traditional herbs and medications purchased without a prescription, [38] participants may have not felt that it was unnecessary to report to health clinics. Although not sexual violence related, a study from Kenya similarly reported that women tended to use traditional herbs and the services of midwives to terminate pregnancies because it was a less expensive option than going to the clinic or hospital. [39] Although the current study did not probe further about disclosure of SVRPs to health care providers, we propose this as an area of future research since it may have implications for the physical and psychological outcomes for the survivor. Additionally, future efforts are needed to increase awareness among prenatal providers to screen for SVRPs, and for sensitivity training around how to handle disclosures of SVRPs. Finally, there is an urgent need to increase access to skilled providers trained in comprehensive post-assault care, including evidence-based methods of pregnancy termination.

### Study Strengths and Limitations

#### Limitations

The qualitative data presented here are derived from a larger sample of participants recruited through respondent-driven sampling (RDS). The results represent only the attitudes and experiences of those interviewed and are not generalizable. Comparison of demographics between the quantitative and the qualitative interviews indicates that married women raising children from SVRPs were under-sampled in the qualitative interviews. It is unknown what other differences in sampling may have existed. Responses were hand recorded, and errors and biases may have occurred in transcription. Some nuances may also have been lost since the responses were not audio-recorded. Translation and interpretation errors may exist in these data, although every effort was made to assure fidelity to the context during translation, analysis and interpretation. Responses may have been influenced by the interviewers’ own biases and perceptions and the presence of the interviewer may have in turn impacted the response. Recall bias among study participants may have also been an issue. Efforts were made, however, to hire and train interviewers who were well versed in qualitative research methods, comfortable with the sensitivities of the subject matter and cultural norms, and knowledgeable in how to probe for more accurate recall. Finally, as with all qualitative research, there may also be interpretation bias by coders and researchers.

#### Strengths

The study also had a number of strengths including accessing a relatively large sample (N = 55) of a hard-to-reach population in an insecure, post-conflict setting. Its mixed methods design also allowed us to draw on knowledge gained in the quantitative data when interpreting the qualitative narratives. The study was also unique in that it offered the opportunity to gain insights on disclosure of SVRPs from both women who carried the pregnancies to term as well as of women who had terminated SVRPs.

## Conclusion

Trust and discretion are the most important factors determining to whom women with SVRPs disclose their pregnancies. The vital role of confidants in giving support after disclosure cannot be overlooked. Providing opportunities for survivors to safely disclose their SVRPs is a necessary first step in allowing them to access safe and comprehensive post-assault care and services. Further research is warranted to better understand why few women disclosed to health care providers in this particular context.

## Supporting Information

S1 File English Qual Child Questionnaire. (DOCX)

S2 File English Qual Term Questionnaire. (DOCX)

S3 File Swahili Qual Child Questionnaire. (DOC)

S4 File Swahili Qual Term Questionnaire. (DOC)
