# Supplementary material for: A Qualitative Analysis of Disclosure Patterns among Women with Sexual Violence-Related Pregnancies in Eastern Democratic Republic of Congo
Source: PLoS One. 2016 Oct 14;11(10):e0164631. doi: 10.1371/journal.pone.0164631 (PMC5065222; doi:10.1371/journal.pone.0164631)
Supplement: S1 File — (DOCX) [file pone.0164631.s001.docx]

My name is __________________. I am a part of a group of researchers trying to better understand what women and children experience when a child is born of sexual violence. We are interested in learning more about your most recent pregnancy that resulted from sexual violence. This interview builds on the survey we conducted with you recently. Your participation is voluntary and you have the option to not answer a question or to end the interview at any time. Your response will be kept confidential and will not be associated with you now or in the future. The discussion will last for approximately one hour. Do you agree to be interviewed?

1. To begin the interview, I would like to ask you about your background. Please tell me about your family. *(probes: How old are you? Are you currently married or do you have a partner? How many times have you been pregnant? How many children do you have? What are their ages? What are their sexes?)*

*Now I would like to discuss the pregnancy you had as a result of sexual violence:*

1. What was your reaction to finding out that you were pregnant as a result of the sexual violence? *(Probes: Can you tell me more about how you felt? What did you do immediately?)*
2. Who did you first tell about the sexual violence related pregnancy and what was their reaction? *(Probes: Why did you choose to tell this person first? What was the reaction of this person? Who else did you talk to about the pregnancy?)*
3. Tell me more about your decision to continue with the pregnancy and to raise this child. *(Probes: What influenced your decision? Did you consider terminating the pregnancy? If yes, why did you decide to continue the pregnancy?)*

*Now I would like to discuss some of your concerns:*

1. What is your greatest concern for yourself?
2. What is your greatest concern for the future of women of DRC?
3. What would be most helpful for children born of sexual violence in DRC?

What would be most helpful for women who become pregnant from sexual violence in DRC?
