# Supplementary material for: A Qualitative Analysis of Disclosure Patterns among Women with Sexual Violence-Related Pregnancies in Eastern Democratic Republic of Congo
Source: PLoS One. 2016 Oct 14;11(10):e0164631. doi: 10.1371/journal.pone.0164631 (PMC5065222; doi:10.1371/journal.pone.0164631)
Supplement: S2 File — (DOCX) [file pone.0164631.s002.docx]

My name is __________________. I am a part of a group of researchers trying to better understand what women experience when they become pregnant from sexual violence. This interview builds on the survey we conducted with you recently. Your participation is voluntary and you have the option to not answer a question or to end the interview at any time. Your response will be kept confidential and will not be associated with you now or in the future. The discussion will last for approximately one hour. Do you agree to be interviewed?

1. To begin the interview, I would like to ask you about your background. Please tell me about your family. *(probes: How old are you? Are you currently married or have a partner? How many times have you been pregnant? How many children do you have? What are their ages? What are their sexes?)*
2. Now I would like to discuss the circumstances of the pregnancy that resulted from the sexual violence. Can you please tell me about the circumstances of the pregnancy? *(Probes: How did you find out that you were pregnant as a result of sexual violence? Did you have any questions about whether the pregnancy was conceived as a result of sexual violence?)*
3. What was your reaction to finding out that you were pregnant as a result of the sexual assault? *(probes: Can you tell me more about how you felt? What did you do immediately?)*
4. Who did you first tell about the rape-related pregnancy and what was their reaction? *(Probes: Why did you choose to tell this person first? What was the reaction of this person? Who else did you talk to about the pregnancy? )*
5. How did you come to a decision to end the pregnancy? *(Probes: Was there anything else that may have influenced your decision? How do you feel about this decision now?)*
6. Did you consider continuing the pregnancy? *(Probes: If yes, what were thoughts on why you wanted to continue with the pregnancy? Would anything have made it easier to continue the pregnancy and to raise the child?)*
7. How did you find out how to end the pregnancy? *(Probes: please explain the process. Who did you go to? What method was used to end the pregnancy? Were you given options on different methods you could use? If so, why did you choose the method you decided to use?)*
8. What made it easy or difficult to obtain a termination? (*Probes: Tell me about the cost of the termination? What are the social, religious, legal issues surrounding termination in your community? What resources/services would have helped you at that time?)*
9. How did you feel emotionally after the termination? *(Probes: What, if any, were your particular emotions? Did your sleep or eating patterns change? Were there social effects from the termination, if so, what?)*
10. What do you think about pregnancy terminations?

*Now I would like to discuss some of your concerns:*

1. What is your greatest concern for yourself?
2. What is your greatest concern for the future of women of DRC?
3. What would be helpful for women who become pregnant from sexual violence in the future?
