# Supplementary material for: A Qualitative Analysis of Disclosure Patterns among Women with Sexual Violence-Related Pregnancies in Eastern Democratic Republic of Congo
Source: PLoS One. 2016 Oct 14;11(10):e0164631. doi: 10.1371/journal.pone.0164631 (PMC5065222; doi:10.1371/journal.pone.0164631)
Supplement: S3 File — (DOC) [file pone.0164631.s003.doc]

**UTAFITI KUHUSU HAFYA YA MWANAMKE WA BUKAVU MWAKA WA**

**UCHUNGUZI KIHALI KUHUSU WATOTO WENYI KUZALIWA KABLA KITENDO CHA UBAKAJI**

Jina langu………………………………………. Niko mumoja wa kundi la watafiti wanaotaka kupata mafasirio kamilifu kuhusu mwanamke na mtoto, mambo wanao ishi wakati mtoto amezaliwa kutokana na ubakaji. Tunapendelea kujua zaidi kuhusu mimba ulio beba kutokana na ubakaji. Hiyi mazungumuzo inatokana na utafiti tuliofanya siku zilio pita. Tunakusihi ujisikie huru kwa kujibu kwa maulizo zetu ao kumaliza maongezi saa yoyote. Hatuta mjulisha mtu wowote kuhusu maongezi yetu. Maongezi yetu ita dumu kiasi ya saa moja. Ikikupendeza, unakubali kujibu kwa maulizo zetu ?

1. Kwa mwanzo ningependa tuzungumuze kuhusu wewe binafsi. Tafazali unaweza kunizungumuzia kwanza kuhusu jamaa lako ?

Ulizo : Una miaka ngapi ? Umekwisha kuolewa ao unaishi tu na rafiki fulani ? Umekwisha kubeba mimba mara ngapi? Una watoto wangapi ? Kila umoja  ana miaka ngapi? Ni wavulana ao wabinti ?

Sasa ikikupendeza, tuzungumuze kuhusu mimba iliyotokana na ubakaji.

2. Ilikuwa je wakati ulitambua kama ulisha beba mimba iliyotokana na ubakaji ?

Ulizo : Unaweza kunifasiriya jinsi ulisikiaka ? Nini umeweza kufanya hapo hapo?

3. Ni nani uliyemjulisha wa kwanza kuhusu ile jambo ya mimba yako iliyotokana na ubakaji, na alisema ao alifanya nini ?

Ulizo : Sababu gani ulimchagua yule mtu ili umujulishe lilo jambo ? Alisema ao kufanya nini ? Nani mwengine uliyemuelezeya hiyo habari ya mimba ?

4. Unielezeye zaidi kuhusu msimamo wako wa kubaki na hiyo mimba na kukomalisha mtoto.

Ulizo : Nini ilituma unakamata pendekezo hiyo?

Iliwezekanaka pia uchukuwe pendekezo la kutoa hiyo mimba kupitiya utashi wako ? Kama divyo, sababu gani ulikusudia kubaki na hiyo mimba ?

Sasa tuzungumuze kuhusu uzuni yako

5. Uzuni yako kubwa kuhusu wewe mwenyewe ni gani?

6. Uzuni yako kubwa kuhusu mwanamke wa Congo siku za usoni ni gani?

7. Ni msaada gani zaidi inaweza kufaliya watoto waliozaliwa kutokana na ubakaji mu Congo ?

8. Ni msaada gani zaidi inaweza kufaliya wanawake wenyi kubeba mimba kutokana na ubakiji mu Congo?
