# Supplementary material for: A Qualitative Analysis of Disclosure Patterns among Women with Sexual Violence-Related Pregnancies in Eastern Democratic Republic of Congo
Source: PLoS One. 2016 Oct 14;11(10):e0164631. doi: 10.1371/journal.pone.0164631 (PMC5065222; doi:10.1371/journal.pone.0164631)
Supplement: S4 File — (DOC) [file pone.0164631.s004.doc]

**UTAFITI KUHUSU HAFYA YA MWANAMKE WA BUKAVU MWAKA WA**

**UCHUNGUZI KIHALI KUHUSU UONDOAJI KIPEKE YA MIMBA**

Jina langu………………………………………. Niko mumoja wa kundi la watafiti wanaotaka kupata mafasirio kamilifu kuhusu mwanamke , mambo wanao ishi wakati mtoto amezaliwa kutokana na ubakaji. Tunapendelea kujua zaidi kuhusu mimba ulio beba kutokana na ubakaji. Hiyi mazungumuzo inatokana na utafiti tuliofanya na wewe siku zilio pita. . Kuhuzuria kwako inafwatana na utashi wako na unauwezo ya kusipo jibu maulizo ama kusimamisha maongezi kwa wakati wowote. Mazungumuzo yetu itawekwa kwa siri na hakuna kitu yoyote itakayo kutambulishwa wewe sasa kama vile siku za usoni. Maongezi yetu ita dumu kiasi ya saa moja. Ikikupendeza, unakubali kujibu kwa maulizo zetu ?

1. Kwa mwanzo ningependa tuzungumuze kuhusu wewe binafsi. Tafazali unaweza kunizungumuzia kwanza kuhusu jamaa lako.

Ulizo : Una miaka ngapi ? Umekwisha kuolewa ao unaishi tu na rafiki fulani ? Umekwisha kubeba mimba mara ngapi? Una watoto wangapi ? Kila umoja  ana miaka ngapi? Ni wavulana ao wabinti ?

2. Sasa ikikupendeza, tuzungumuze kuhusu mambo iliyo sababisha mimba iliyotokana na ubakaji. Tafazali uniambiye umebeba hiyo mimba je.

Ulizo : Ulijuwa je kama hiyo mimba ilitokana na ubakaji ? Umejiuliza ao walikuuliza kama ile mimba ilitokana na ubakaji ?

3. Umefanya nini wakati ulipo tambua kama hiyo mimba ilitokana na ubakaji ?

Ulizo : Unaweza kuniambiya zaidi ngisi ulisikiyaka? Ulifanyaka nini hapo hapo?

4. Ni nani ulijulisha wa kwanza kuhusu ile jambo ya mimba kutokana na ubakaji ?, na alisema ao amefanya nini ?

Ulizo : Sababu gani ume muchaguwa peke yake? Alisema ao amefanya nini? Nani mwengine ulimuelezeya habari hiyo ya mimba?

5. Umeamuwa je kutowa hiyo mimba?

Ulizo : Kuna kitu ingine imekusukuma ku kamata hiyo uamzi ? Kwa sasa unasikiya je juu ya hiyo uamzi?

6. Umeamuwa binafsi kukubali kubaki na hiyo mimba ?

Ulizo: Kama ndivyo, sababu gani uliamuwa kukubali kubaki na hiyo mimba? Kuna sababu ao kitu iliyo tuma una baki na hiyo mimba hadi kuzaa na kumleya huyo mtoto?

7. Ulifaulu je kwa kutowa ile mimba?

Ulizo : Tafazali ufasiriye umefanya ni kwa kutowa ile mimba. Ulimuona nani ? Alitumiya njia gani nini kwa kutowa ile mimba ? Umekuwa huru kwa kuchaguwa ngisi ya kutowa ile mimba ? Kama ndiyo, fasiriya sababu gani umecaguwa njia ile  kwa kuitowa?

1. Nini imekusaidiya ao kukuzuwiya kwa kutowa ile mimba ?

Ulizo : Unijulishe umelipa nini kwa kutowa ile mimba. Kumekuwa shida na majirani ama wengine, kanisa, sheria kuhusu kutowa mimba hiyo? Umepata msaada kutoka wapi kwa muda huyo ? Umepata msaada wa namna gani kwa ile wakati ?

1. Umejisikiya namna gani kindani kisha kutowa mimba ?

Ulizo : Umejisikiya namna gani kwa kweli ndani ya roho yako ?  ? Namna yako ya kusizia ama kukula imebadirika ? Kutowa mimba imeleta mabadriko katika maisha yako na majirani ama watu wengine, kama ndiyo, ni gani?

10. Unawaza nini kuhusu kutowa mimba ?

Sasa tuzungumuze kuhusu uzuni uliyokuwa nayo .

11. Uzuni yako kubwa kuhusu wewe mwenyewe ni gani?

12. Uzuni yako kubwa kuhusu mwanamke wa Congo siku za usoni ni gani?

13. Kwa siku za usoni,ni msaada gani inaweza kufaliya wanawake wenyi kubeba mimba kutokana na ubakiji mu Congo?
